# Molecular Effects of Mutations in Human Genetic Diseases

**DOI:** 10.3390/ijms23126408

**Published:** 2022-06-08

**Authors:** Emanuela Leonardi, Castrense Savojardo, Giovanni Minervini

**Affiliations:** 1 Department of Woman and Child Health, University of Padova, 35128 Padova, Italy; 2 Pediatric Research Institute, Città della Speranza, 35127 Padova, Italy; 3 Dipartimento di Farmacia e Biotecnologie, University of Bologna, 40126 Bologna, Italy; castrense.savojardo2@unibo.it; 4 Department of Biomedical Science, University of Padova, 35131 Padova, Italy; giovanni.minervini@unipd.it

Next-generation sequencing (NGS) has enormously improved the identification of disease-candidate genetic variants. However, most NGS studies did not tackle the functional interpretation of these variants to recognize a new disease gene or to categorise clinically relevant variants. Moreover, knowledge about the molecular effects of causal mutations, emerging at the interface of human genetics, computational biology, molecular biology, and biophysics, may provide insights into pathogenic mechanisms underlying diseases that can be targeted to develop novel therapeutic strategies.

This Special Issue (SI) aimed to attract publications highlighting the currently available computational and experimental approaches to investigate molecular effects of genetic mutations. The eight papers published in this SI described case studies that may provide a useful framework for understanding the general molecular defects underlying a broad and diverse spectrum of human diseases. These included multifactorial or polygenic disorders, such as lung cancer (OMIM*612052) and osteoporosis (OMIM*166710) [1,2], or monogenic disorders, such as Long QT Syndrome 1 (LQT; OMIM*192500), Hyaline fibromatosis syndrome (OMIM*228600), Dystrophic epidermolysis bullosa (RDEB; OMIM*226600), *Xeroderma Pigmentosum* C Phenotype (XP-C; OMIM*278720), and MTHFR deficiency (OMIM*236250) [3,4,5,6,7].

Studying the effects of a genetic variant can have several specific purposes, for instance, the following: (i) evaluating the pathogenicity of a novel variant; (ii) identifying the molecular mechanisms supporting the association of the variants with a disease or with the severity of the disease; (iii) investigating the differential effects of some variants associated with distinct phenotypic expressions of the disease. Two of the SI-published works investigated the role of novel genetic variants in the ANTXR2 and LRP5 genes identified by NGS of affected individuals from selected cohorts [2,4]. Diabasana et al. studied the molecular effects of a small nucleotide polymorphism (SNP) of CHRNA5 gene, known to confer susceptibility for lung disease [1]. Beilin et al. investigated the correlation between specific mutations, selected among hundreds of COL7A1 reported variants, and the varying severity of cutaneous manifestation [5]. Oertly et al. investigated a single loss of function (LoF) variant in KCNQ1 associated with an atypical autosomal recessive LQT trait [3]. To highlight possible mechanisms of protein destabilisation due to residue change, Savojardo et al. explored the structural impact of all 72 reported disease-causing variants of the MTHFR gene [8]. In other cases, as that of Kobaisi et al., the studied variant depends on the available patient-derived cells to be used as a model system for a specific disease [7].

Prior to functional studies, due to time-consuming and economical high costs of these experiments, variant pathogenicity can be assessed by computational methods. Multiple sequence- and structure-based tools have been developed to estimate the deleteriousness of genetic variants. In the work of Petrosino et al., the performance of state-of-the-art methods for predicting protein stability change and pathogenicity was assessed on a set of 164 cancer-related variants, whose effects on protein stability and function were experimentally determined. Their work suggests that the integration of experimental and computational approaches is crucial to reach significant gains in the prediction of the phenotypic impacts of genomic variations [6]. For instance, Rocca et al. used both sequence and structure analysis, together with pathogenicity and stability predictors to investigate the putative pathogenic role of novel genetic variants identified in a cohort of 128 males with idiopathic low bone mass [2]. Using their results paired with experimental data from literature, they developed an integrated Bayesian classification model to calculate the pathogenicity risk of these variants [2]. However, the direct inspection of protein sequence, structure, and evolutionary conservation can be used to extract various physicochemical properties highlighting possible molecular mechanisms of protein alteration due to residue change. Savojardo et al. used the MTHFR solved structure to correlate solvent exposure, occurrence of protein–protein interaction sites, and protein destabilization with the annotation of being a disease-associated variant. In this way, they showed that most of the disease-related variants affect the stability of the protein, including the mutated positions at the homodimer interface [8]. Moreover, molecular dynamic simulation has been widely used to predict the effects of genetic variants on the protein structure and function. Rocca et al. applied dynamic simulation to investigate possible structural arrangements induced by the two LRP5 variants classified as pathogenic [2].

The impact of a genetic variant can be experimentally investigated at multiple levels and depends on the studied gene; in particular, it depends on the molecular function of the encoded protein, the biological process involved in the disease, and on the tissue where the gene is expressed. A specific protein function and the pathway in which it is involved can be tested by integrating both in in vivo and in vitro approaches, including mammalian models or more simple organismal models, as well as patient-derived systems, such as patient-specific cell lines, human-induced pluripotent stem cells and organoids. A number of papers published in this SI exploited the potential of this mixed approach employing protein-specific assays to validate the pathogenicity of the target variant(s). By means of electrode voltage clamp experiments, Oertly et al. characterised the electrophysiological properties of the voltage-gated K+ channel KCNQ1 mutant, which was expressed in Xenopus laevis oocytes and compared to oocytes injected with KCNQ1 wild-type [3]. In the study of Jorik M. van Rijn, the anthrax-toxin assay was performed in patient-derived and control intestinal organoids, to confirm the impact of the p.Ser52Phe missense variant on the physiological ANTXR2 function [4]. Due to the rarity of the disorder and the difficulties to obtain specimens from patients, the authors used the homology-directed repair-guided gene editing CRISPR/Cas9 approach to create ANTXR2 knock-out (KO) organoids. In this case, the anthrax-toxin assay was applied to select ANTXR2 KO organoids [4]. Another example of a protein-specific test is provided by Kobaisi et al., who evaluated the expression of XPC, a DNA damage sensor protein, and measured the photosensitivity, and the ability to repair UltraViolet B radiation (UVB)-induced lesions of XP-C cells immortalised from patient primary fibroblasts. In this case, the readout of the protein function were the photoproducts generated after UVB exposure and measured by LC-MS/MS in the DNA extracted from XP-C cells [7].

Direct inspection of patient-derived tissues can be used to identify abnormal molecular and morphological features in the patients compared to healthy individuals. In the work of Diabasane et al., lung tissue sections from patients carrying the CHRNA5 SNP (rs16969968) were used to assess its impact on epithelial remodelling in human lungs [1]. The significant decrease in multiciliated cells in bronchial epithelia of SNP patients compared to WT patients suggested a SNP-associated alteration of ciliogenesis [1]. In another SI work, Jorik M. van Rijn et al. used second harmonic generation (SHG) imaging, which visualises non-labelled fibrillar proteins such as collagen, and electron microscopy inspection of duodenum and skin sections from ANTXR2-deficient patients [4]. They demonstrated that loss of ANTXR2-mediated signalling yields abnormal extracellular matrix composition paired with significant collagen VI accumulation in the duodenum [4]. Beilin et al. used skin biopsies from patients with Recessive Dystrophic Epidermolysis Bullosa (RDEB) to study, by means of immunohistochemistry (IHC), the intensity and localization of type VII collagen (COL7A1) expression [5]. In addition, fibroblast markers’ expression, including those of COL7A1, were quantified by immunocytochemistry (ICC) using patient-specific RDEB fibroblast lines [5]. They also characterised and compared the morphological properties of primary dermal fibroblast cultures from RDEB against healthy patients by using forward (FSC) and side (SSC) scatter from flow cytometry data [5].

Lastly, the so called ‘omics’ approaches and bioinformatics network analysis could complement and integrate the experimental findings allowing a more exhaustive comprehension of the molecular mechanisms underlying genetic diseases. For instance, transcriptomic data from RDEB fibroblasts have been used to explain the morphological properties and altered processes observed in RDEB fibroblasts [5]. The network and gene ontology analysis of the differentially expressed genes (DEGs) pointed toward specific genes encoding factors directly involved in inflammation and ECM organisation, associated with pro- and antifibrotic changes, or with cell ageing supporting the traits of senescence observed in the cells. Furthermore, the different pattern of DEGs, although influenced by the individual genetic background, can explain the association of different COL7A1 mutations with the severity of the clinical manifestations [5].

Once the molecular mechanisms affected by a genetic variant have been delineated, possible therapeutic interventions can be proposed. Simply, the findings of Beilin et al. that fibroblasts from RDEB patients with specific COL7A1 mutations presented extremely low level of type VII collagen suggest that these patients could be treated with infusion of recombinant COL7A1, skin engraftment or with bone-marrow transplantation treatments [5]. Furthermore, the molecular or morphological properties observed in the targeted model system can be used to systematically test an array of chemicals to rescue the observed pathological phenotype. This SI includes the work of Kobaisi et al., who performed a high-throughput screen to identify chemicals capable of ameliorate the hyper-photosensitivity and accumulation of photoproducts observed in XPC-mutated cells [7].

Overall, the presented SI provides to readers with a wide range of studies, adopting diverse approaches ranging from computational to experimental methods, to elucidate and investigate molecular effects of mutations at different levels and scales.
